# Adverse drug reaction profiles of histone deacetylase inhibitors

**DOI:** 10.1038/s41598-025-19717-w

**Published:** 2025-10-14

**Authors:** Ruqayyah Begum, Jason L. Parsons, Alan M. Jones

**Affiliations:** 1 School of Pharmacy, School of Health Sciences, College of Medicine and Health, University of Birmingham, Edgbaston, Birmingham, B15 2TT UK; 2 Department of Cancer and Genomic Sciences, University of Birmingham, Edgbaston, Birmingham, B15 2TT UK

**Keywords:** Histone deacetylase inhibitor, Adverse drug reaction, World health organisation, VigiAccess, Polypharmacology, Pharmacovigilance, Health care, Public health, Epidemiology

## Abstract

The safety and tolerability of histone deacetylase inhibitors (HDACIs) were always a matter of concern. This study aimed to explore the global observational adverse drug reaction (ADR) profiles of the HDACIs: vorinostat, belinostat, panobinostat, pracinostat, entinostat, romidepsin, bufexamac and sodium phenylbutyrate. This study focussed on the investigation whether associations between HDACIs’ ADR profiles and their physicochemical and pharmacological properties exist and how these associations may be harnessed to contribute to safer clinical use. The ADRs of HDACIs were curated from the World Health Organisation (WHO) VigiAccess database (1976–2024). Pharmacology data was curated from the chemical database of bioactive molecules with drug-like properties, European Molecular Biology Laboratory (ChEMBL). Physiochemical and pharmacokinetic properties were curated from PubChem, Drug Bank, ChemDraw and FDA new drug application documents for the HDACIs studied. In total, *n* = 12,779 ADRs were reported for the selected HDACIs, with *n* = 15/27 of the system organ class (SOC)-related ADRs showing statistical significance (*X*^*2*^, *P* < .05). Vorinostat accounted for *n* = 4,225 of the total ADRs, which may be explained by (1) its extensive use in the clinic and (2) it possessed the most clinically achievable off-target pharmacological interactions (*n* = 9). This included potent inhibition of the human-ether-à-go-go-related gene (*h*ERG) ion-channel (IC_50_ = 322 nM vs. c_max_ = 1,200 nM), which is likely a contributing factor to the highest rate of cardiac ADRs observed (*n* = 146, *P* < .05). Musculoskeletal ADRs associated with vorinostat (*n* = 58, *P* < .05) can be ascribed to its unique inhibition of HDAC4 (IC_50_ = 540 nM vs. c_max_ = 1,200 nM). The gastrointestinal (GI) ADRs of panobinostat (*n* = 488, *P* < .05*)* can partly be related to HDAC3 inhibition (IC_50_ = 2.0 nM vs. C_max_ = 8.0 nM). The absence of HDAC9 inhibition was unique to entinostat and may explain its beneficial drug reaction in depression (*n* = 0 cases). Thrombocytopenia observed with vorinostat, panobinostat and romidepsin may stem from dual inhibition of HDAC1 and HDAC2 and their higher volume of distributions (V_d_). Despite all HDACIs having a similar mechanism of action, their unique pharmacology and differing physicochemical and pharmacokinetic properties landscape results in varying ADR profiles.

**Supplementary Information:**

The online version contains supplementary material available at 10.1038/s41598-025-19717-w.

## Introduction

Adverse drug reactions (ADRs) refer to unintended responses to a drug related to any dose, and account for up to 5% of hospital admissions worldwide, at a cost to the Europe Union of 79 Bn EUR *per annum*^1,2^. ADRs can be classified by the DoTS and Rawlin-Thompson guidelines (Table S1)^3,4^. ADRs continue to be a challenge for healthcare systems, often prolonging hospital stays and complicating treatment^5^ and contribute to or cause 1 in 5 hospital admissions of patients with cancer^6,7^.

Histone deacetylase inhibitors (HDACIs) are a class of drugs, used to treat diseases, including cancer^8,9^. Histone deacetylase (HDAC) enzymes control gene expression and can change the acetylation state of histone proteins, which are responsible for organising DNA in the nucleus^10^. HDAC enzymes participate in several biological processes including cell development, differentiation, proliferation, and apoptosis^11^. There are 18 HDAC enzymes which can be grouped into 4 major classes^12^. Class I (HDAC1, 2, 3 and 8) are typically found in the nucleus, class IIa (HDAC4, 5, 7 and 9) and class IIb (HDAC6 and 10) typically shuttle between the nucleus and cytoplasm, and class IV (HDAC11) is typically found in the nucleus. HDACs belonging to classes I, II and IV are zinc-dependent enzymes, and are often referred to as the classical HDACs^12,13^. Whereas the class III HDACs (includes sirtuins 1–7) are structurally distinct and are nicotinamide adenine dinucleotide (NAD+) dependent enzymes^13^.

**Fig. 1:**
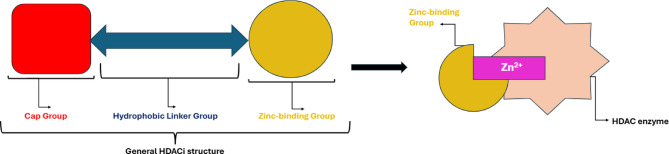
The general structure for HDACIs; *colour coding; red square for cap group*,* blue arrow for hydrophobic linker group and yellow circle for zinc-binding group.* The zinc-binding group chelates the Zn^2+^ within the HDAC enzyme active site, inhibiting HDAC activity.

HDACIs have three key structural elements: a Zinc-binding group (ZBG), a cap group and a hydrophobic linker chain (Fig. 1 and Figure S1)^14,15^. The ZBG of the HDACI interacts with Zn^2+^ within the HDAC deacetylase domain, disrupting enzyme activity^8,15^. HDACIs can be grouped based on their chemical structures and their type of ZBG^15^. Hydroxamic acid HDACIs have a hydroxamate ZBG, benzamide derivatives have a distinct benzamide ZBG, cyclic peptides bind to Zn^2+^ via a thiol functional group and short-chain fatty acids derivatives feature a carboxyl ZBG^16,17^. Generally, hydroxamic acid derivatives are amongst the more potent HDACIs with fast-on/fast-off kinetics, whereas benzamides are considered less potent with slower binding kinetics^18^. Both function as bidentate Zn^2+^ binders^18^. Cyclic peptides bind moderately to Zn^2+^, in a monodentate manner and short-chain fatty acids typically have the weakest zinc-binding interactions^14,19^.

Results from clinical trials have demonstrated the effectiveness of HDACIs, resulting in licensing across multiple jurisdictions^13,18,20–24^ (Table S2). This includes the phase II BELIEF trial which established a 26% overall response rate, with 13 complete responses, in patients taking belinostat monotherapy for refractory peripheral T-cell lymphomas^25^. The PANOROMA-1 phase III trial showed that adding panobinostat to dexamethasone and bortezomib increased median overall survival to 40 months versus 36 months for a placebo, or dexamethasone and bortezomib in patients with refractory multiple myeloma^26^. Belinostat and panobinostat were subsequently approved by the FDA in 2014 and 2015, respectively.

However, pan-HDACI’s, for example sodium valproate, underscore a potential challenge of non-selective HDAC inhibition amongst other potential mechanisms of action (MoA). Pan-HDACIs are non-selective, targeting multiple HDAC isoforms, which can result in ADRs^27,28^. Sodium valproate is associated with side effects, including hepatotoxicity, osteoporosis, and neurotoxicity^29^. Most concerning is its teratogenicity, and its associations with congenital and developmental ADRs^30^. Thus, women of child-bearing age, prescribed sodium valproate are now enrolled on a pregnancy prevention plan (PPP) in the United Kingdom^31^. Approximately 20,000 babies in the United Kingdom have been born with foetal valproate syndrome, and 1 in 10 babies were born with *spina bifida* after *in utero* exposure to sodium valproate^32,33^. The challenges of treatment with pan-HDACIs are compounded by the fact that many cancers overexpress specific HDAC isoforms. For example, HDAC1, 2 and 6 are overexpressed in peripheral T-cell lymphoma and HDAC5 and 7 in acute-myeloid leukaemia^34,35^. Therefore, HDAC inhibition therapy continues to be a promising strategy to improve benefit vs. risk profile in these patients^36^.

Clinical trials have revealed the main side effects linked to using HDACIs are myelosuppression, cardiotoxicity, and diarrhoea^37^. However, there is a limited understanding of their ADR profiles beyond carefully controlled clinical trials; in a ‘real-world’-setting both patient and environmental factors are often more varied and complex. A comprehensive understanding of the toxicities associated with HDACIs is crucial to ensure their safe use in clinical practice.

Underpinning this requires a clear understanding of all 18 HDAC isoforms. Though, some isoforms in particular HDAC1 and 2 have been more extensively studied than others (HDAC 3–8, 11–18)^38–40^. There are few HDAC9 selective inhibitors in clinical trials, and the role of HDAC9 during tumorigenesis is still largely unclear^41^. Similarly, few published studies have focused on developing HDAC10 selective inhibitors, despite the growing understanding of its role in polyamine deacetylase and thus drug-resistant cancer biology^42^. The HDAC selective inhibitor RGFP109 has shown potential for temozolomide resistance in glioblastoma cell lines in preclinical trials, though no advance to clinical trials have been made at the time of writing^43^. Therefore, there is an ongoing need to focus research on HDACIs, as part of a broader effort to develop effective therapies that are also better tolerated by patients.

The majority of HDACIs are not licensed in the United Kingdom, suggesting concerns remain relating to their safety, tolerability and efficacy (Table S2). Identifying causal links between HDAC isoforms and ADRs could facilitate the development of more selective HDACIs, improving patient safety and therapeutic outcomes. An extensive literature search revealed a single pharmacovigilance study using the FDA Adverse Event Reporting System (FAERS) on 6 HDACIs^44^. Herein, this study will be the first to examine global real-world data using the WHO VigiAccess dataset to investigate the pharmacovigilance of all HDACIs and ADRs resulting. It will offer insights into the safety of HDACIs from a global standpoint to facilitate their safer use in clinical practice.

This study will focus specifically on 8 HDACIs; vorinostat, belinostat, panobinostat, pracinostat, entinostat, romidepsin, bufexamac and sodium phenylbutyrate^13,18,20,22–26,37^ (Table S3).

## Aims and objectives

- To explore the ADR data of the HDACIs vorinostat, belinostat, panobinostat, pracinostat, entinostat, romidepsin, bufexamac and sodium phenylbutyrate using the WHO VigiAccess database.
- Determine the unique physicochemical and pharmacological properties for each HDACI studied.
- Determine links between the ADR profiles of these HDACIs and their physicochemical and pharmacological properties.
- Postulate potential mechanisms to rationalise any side effect differences.

## Methods

The HDACIs, vorinostat, belinostat, panobinostat, pracinostat, entinostat, sodium phenylbutyrate, romidepsin and bufexamac were studied based on the inclusion and exclusion criteria of this research (Table 1 and Table S4).

**Table 1:** Inclusion and exclusion criteria for this study, to select the HDAC inhibitors.

Inclusion criteria	Exclusion criteria
Drugs with known HDAC isoform inhibition.	Drugs with no known HDAC isoform inhibition.
Available ADR data on the WHO VigiAccess database, minimum number of reports (*n* = 5).	No ADR data available on the WHO VigiAccess database.
> 5 ADRs in a single system organ class.	< 5 ADRs in every system organ class.
A drug currently prescribed, in clinical trials or withdrawn from the market.	Supplements or amino acids.

### Physiochemical properties

The physicochemical parameters calculated were molecular weight (Da), total polar surface area (^*t*^PSA), p*K*_a_, hydrogen bond donors (HBDs), hydrogen bond acceptors (HBAs), cLog_10_P, cLog_10_D^7.4^ and if the drug was a p-glycoprotein substrate. Calculated molecular weight, ^*t*^PSA and cLog_10_P were calculated using Perkin Elmer ChemDraw version 22.0. The p*K*_a_, HBDs, HBAs and whether the HDACIs were p-glycoprotein substrates were curated and analysed using Drug Bank data searches and cross-validated with primary literature^45^. cLog_10_D^7.4^ was curated from the chemical database of bioactive molecules with drug-like properties, European Molecular Biology Laboratory (ChEMBL)^46^. pIC_50_ was determined by the negative log_10_ of the median IC_50_ of its single *homosapien* protein target. Lipophilic Ligand Efficiency (LLE) was calculated using the following formula: pIC_50_ – log_10_P. An LLE > 5 is linked to fewer off-target interactions thus reducing the risk of ADRs^47^.

Selected physicochemical properties increase penetration through the blood-brain barrier (BBB), specifically: a molecular weight of < 450 Da, < 6 HBD, < 2 HBA, a neutral or basic drug, ^*t*^PSA of < 90 Å^2^, cLog_10_D^7.4^ between 1 and 3, and not a p-glycoprotein substrate. Risk of penetration of the BBB increases with the more requirements met^48^.

### Pharmacokinetic properties

The pharmacokinetic properties calculated were C_max_ (peak plasma concentration), T_max_ (time to reach peak plasma concentration), CYP_450_ isoform metabolism where applicable, total clearance, half-life (t_1/2_), volume of distribution (V_d_) and plasma protein binding (PPB). C_max_ values were curated from FDA monographs for vorinostat, romidepsin and sodium phenylbutyrate and converted to nM^49–51^. PPB and CYP_450_ isoform inhibition was gathered from the Drug Bank, Electronic Medicines Compendium (EMC), and PubChem^45,52,53^. The remaining pharmacokinetic data was curated using Boolean logic in the Reaxys and Web of Science databases by searching the ‘drug name’ together with the ‘parameter required’^54–58^. To the best of our knowledge and after extensive literature searches, no C_max_ for bufexamac has been reported (Table 2).

### Target affinity

The ChEMBL database was used to collect all reported bioactivity data for single homosapien protein targets for each HDACI. A median IC_50_ data point was calculated if a *single* homosapien protein target had > 1 IC_50_ value reported under analogous experimental conditions^46^. This was performed by calculating the pIC_50_ using the following formula: pIC_50_ = -log_10_(IC_50_ in Molar units).

### ADR reporting

ADR data was retrieved from the World Health Organisation (WHO) VigiAccess data set (https://www.vigiaccess.org/) on the 14.11.24 for the HDACIs studied^59^. ADRs reported for vorinostat (2006-), belinostat (2008-), panobinostat (2009-), pracinostat (2015–2022), entinostat (2011–2023), sodium phenylbutyrate (2002-), romidepsin (2009-) and bufexamac (1976–2022) were extracted, cleaned, and analysed using Excel version 24.11 for Microsoft 365.

### Statistical analysis

Excel version 24.11 for Microsoft 365 was used to perform the chi-squared tests on the ADR data for each system organ class (SOC), to determine the statistical significance of the ADR signal (*P* <.05). ADR at the SOC-level (where reports, *n* < 5) were excluded from the chi-squared test^60^.

### Ethical approval

Ethical approval by the School of Pharmacy sub-ethics committee was not required, as all the data retrieved is publicly available and fully anonymised.

## Results

**Table 2:** Summary of the physicochemical, BBB penetration and pharmacokinetic properties for the eight HDACIs studied.

Variable	Vorinostat	Belinostat	Panobinostat	Pracinostat	Entinostat	Romidepsin	Sodium phenylbutyrate	Bufexamac
**Molecular obesity and on-target efficiency metrics**								
cLog_10_P	0.90	1.85	1.85	3.21	3.21	3.44	−1.72	1.85
pIC50	7.48	7.75	8.68	7.20	9.30	9.52	4.19	4.45
LLE	6.58	5.90	6.83	3.99	6.09	6.08	5.91	2.60
**BBB penetration properties**								
Mw (Da)	264.32	318.35	349.43	358.48	376.41	540.69	186.18	223.27
HBDs	3	3	4	2	3	4	0	2
HBAs	3	4	3	4	4	5	2	3
p*K*_a_	8.91 (acid)	7.82 (acid)	9.31 (acid)	8.85 (acid)	4.74 (base)	10.67 (acid)	4.66 (base)	8.86 (acid)
^*T*^PSA (Å)	78.43	95.50	73.39	68.17	105.81	142.70	40.13	58.56
cLog_10_D^7.4^	1.99	1.69	0.97	1.09	2.31	1.08	−0.04	1.95
P-glycoprotein substrate	No	Yes	Yes	No	No	Yes	No	No
No. of BBB requirements met	5	3	4	5	5	2	5	5
**Penetration through the CSF?**	Yes	Limited	Limited	n.d.	n.d.	Limited	Yes	n.d.
**Pharmacokinetics properties**								
t_1/2_ (h)	2	1.10	30	6.80	52	3	0.77	1–2
T_max_ (h)	4	0.42	2	1.80	1	4	1.35	-
C_max_ (nM)	1,200	220	8.0	940	49	204	1,200	n.r.
CYP_450_ metabolism	Negligible	2A6, 2C9, 3A4	3A4, 2D6	1A2, 3A4	Unknown	3A4	1A1, 1A2, 1B1, 2E1	Negligible
V_d_ (L)	150	409	27.20	3.50	1.36	44.50	0.20	-
Clearence (L/hr)	150	74.4	28.6	9.2	14.9	8.4	16	-
PPB (%)	71	95.80	90	94	94	94	98	-

LLE, lipophilic ligand efficiency; BBB, blood brain barrier; Mw, molecular weight; HBDs, hydrogen bond donors; HBAs, hydrogen bond acceptors; p*K*_a,_ the acid dissociation constant; ^*t*^PSA, total polar surface area; cLog_10_D^7.4^, calculated Log_10_D at pH 7.4; CSF, cerebrospinal fluid; t_1/2,_, half-life; T_max_, time taken to reach peak plasma concentration; C_max,_ peak plasma concentration achieved; CYP_450,_ Cytochrome p450; V_d,_ volume of distribution; PPB, plasma protein binding; n.d., not determined, n.r., not reported.

### Chemical data

The chemical data for the HDACIs is displayed in Table 2. The most lipophilic drug was romidepsin (cLog_10_*P* = 3.44) and was also the most potent (pIC_50_ = 9.52). Pracinostat and bufexamac had LLEs < 5, (3.99 and 2.60 respectively); this suggests they may have more off-target interactions.

Vorinostat, pracinostat, entinostat, sodium phenylbutyrate and bufexamac are the most likely to penetrate the BBB, having all met 5/7 requirements. Though all the HDACIs had < 6 HBDs, none met the > 2 HBAs limit. Only entinostat and sodium phenylbutyrate were bases, the rest were acids. The ^*t*^PSA of belinostat (95.50), entinostat (105.81) and romidepsin (142.7), all exceeded > 90Å. The cLog_10_D^7.4^ of sodium phenylbutyrate (−0.04) was the only one outside the desired range of 1–3, correlating with its notably low V_d_ (0.2 L). Romidepsin was the heaviest HDACI (540.69 Da), with a molecular weight > 450 Da and overall was the least likely to penetrate the BBB, meeting only 1/7 requirements. This aligns with cerebrospinal (CSF) measurements in primates which showed romidpesin CSF exposure was 2% after IV administration^61^.

Entinostat had the longest half-life (52 h), reflected in its once weekly dosing, whereas belinostat had the shortest (1.1 h) which is dosed daily (Table 2). Romidepsin and vorinostat both had the highest T_max_ (4 h), whereas belinostat was the lowest 0.42 h. Sodium phenylbutyrate had the highest PPB (98%) compared to vorinostat with the least (72%).

**Table 3:**
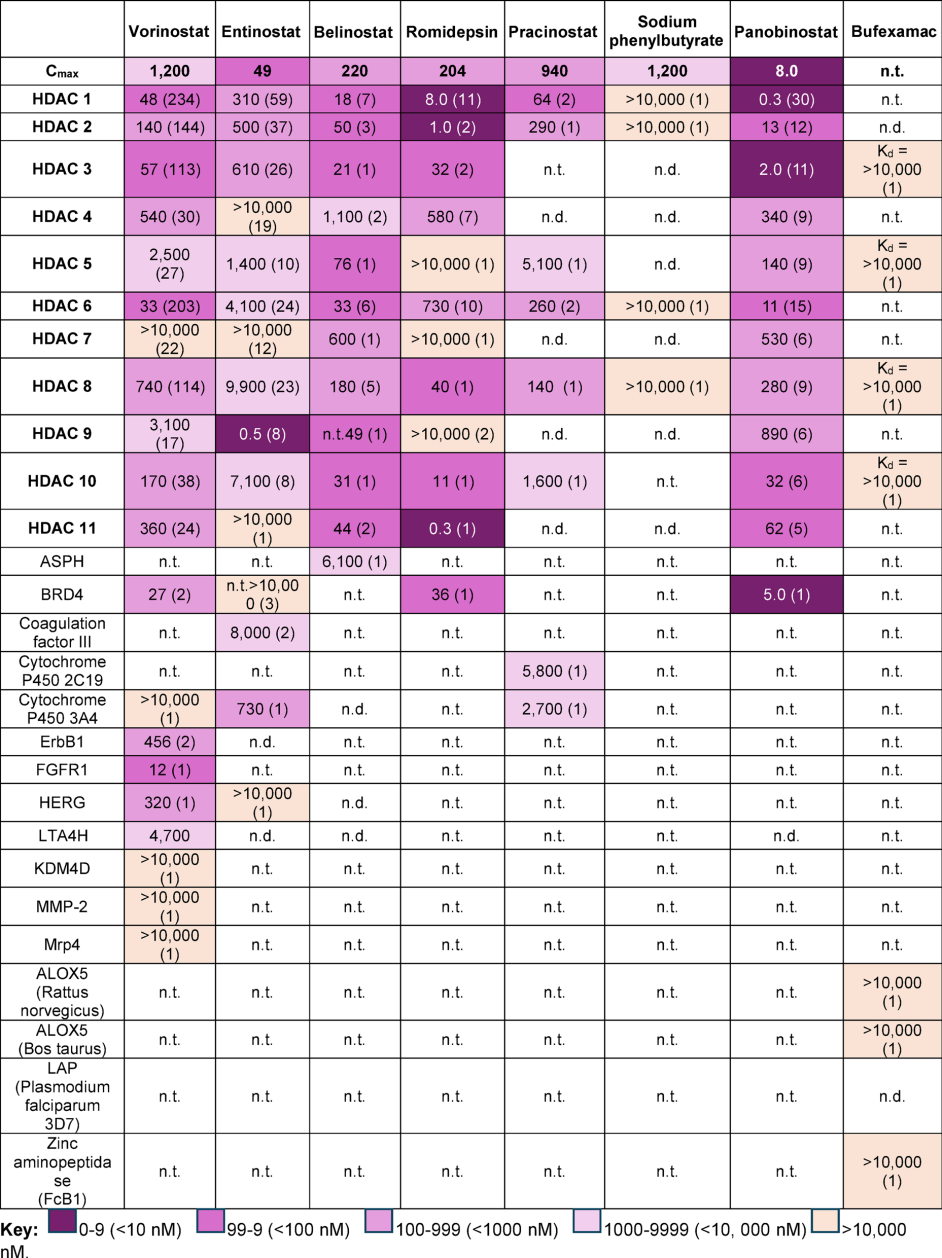
Summary of the pharmacology data for the eight HDACIs studied displayed as the median IC_50_ values (nM) for each single homosapien protein target alongside their clinical C_max_ (nM) unless specified otherwise. Selected potency values for bufexamac are displayed as K_d_.

### Target affinity

The pharmacological data for the HDACIs are displayed in Table 3. Panobinostat was the most potent towards HDAC1 (0.3 nM), HDAC2 (13 nM) and HDAC3 (2.0 nM) vs. C_max_=8 nM. Panobinostat, vorinostat and romidepsin had additional, clinically achievable, off-target interactions with BRD4, though panobinostat was the most potent (5.0 nM vs. 27 nM vs. 36 nM, respectively). Entinostat uniquely potently inhibited HDAC9 (0.5 nM vs. C_max_=49 nM), but potential off-target interactions with coagulation-factor III and CYP450 3A4(8,000 nM and 730 nM, respectively) were above the C_max_. Similarly, belinostat was the only potent inhibitor of HDAC5 (76 nM vs. C_max_=220 nM) but potential off-target interaction with ASPH were above the C_max_. Pracinostat was able to clinically inhibit HDAC1 (64 nM), HDAC2 (290 nM) and HDAC6 (260 nM) vs. C_max_ =940 nM, but potential off-target interactions (*n* = 2) were above the C_max_. The pan-HDACI vorinostat showed the greatest number of off-target interactions (*n* = 9), though only (*n* = 4) were clinically achievable with the most potent being for FGFR1 (IC_50_ = 12 nM vs. C_max_=1,200 nM). Sodium phenylbutyrate did not clinically inhibit any proteins based on its C_max_ (IC_50_ > C_max_), and no C_max_ was reported for bufexamac.

**Fig. 2:**
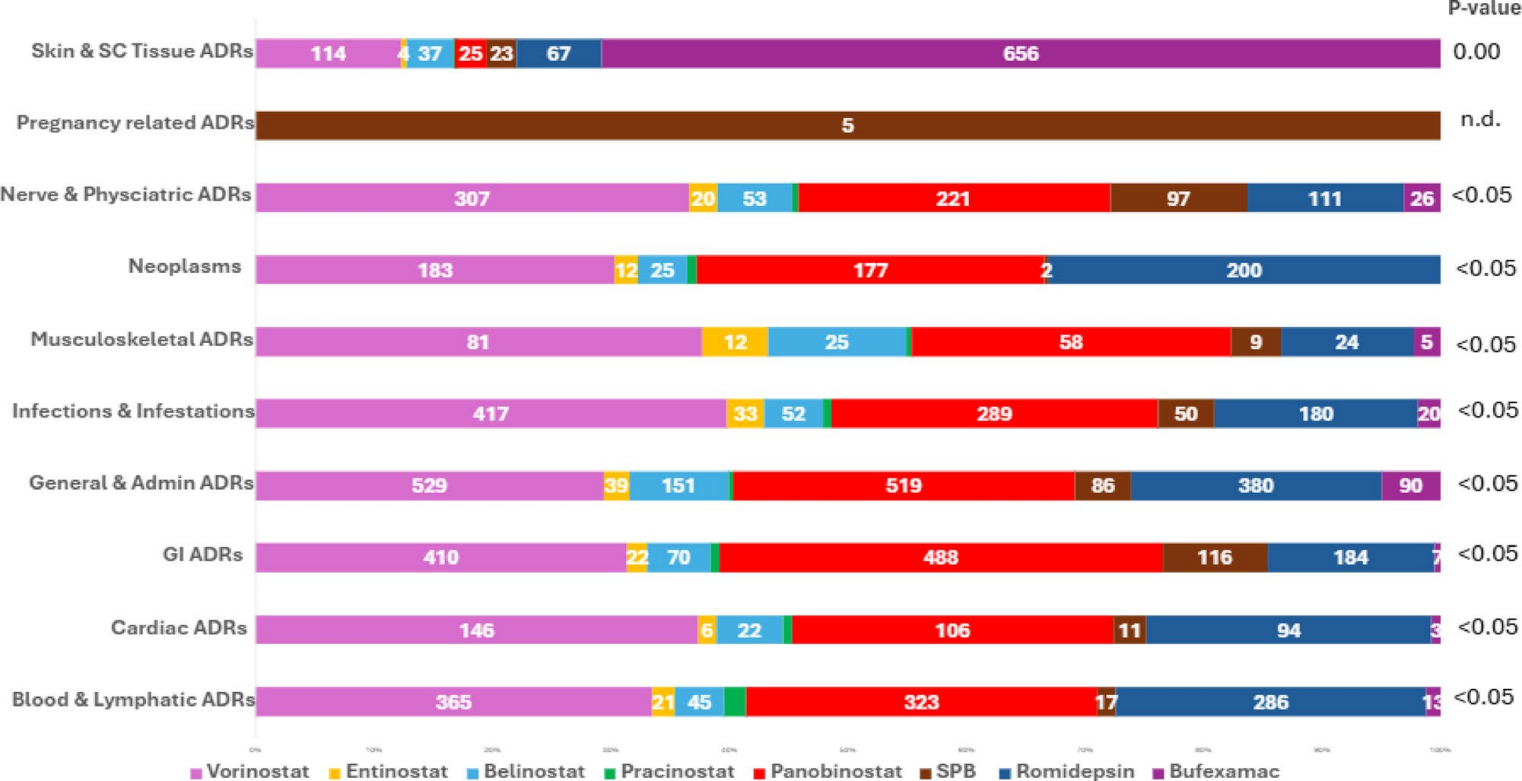
A stacked bar chart representing the number of ADRs across the main system organ classes for the HDAC inhibitors studied, alongside the *P*-value for each SOC. Data for pracinostat was omitted due to the low number of reported ADRs; the full breakdown of ADR profiles, including pracinostat, is available in Table S5. Abbreviations: SC, subcutaneous; Pregnancy related ADRs, pregnancy, puerperium & perinatal ADRs; Admin, Administration; GI, gastrointestinal; *P*-value, probability value.

**Fig. 3:**
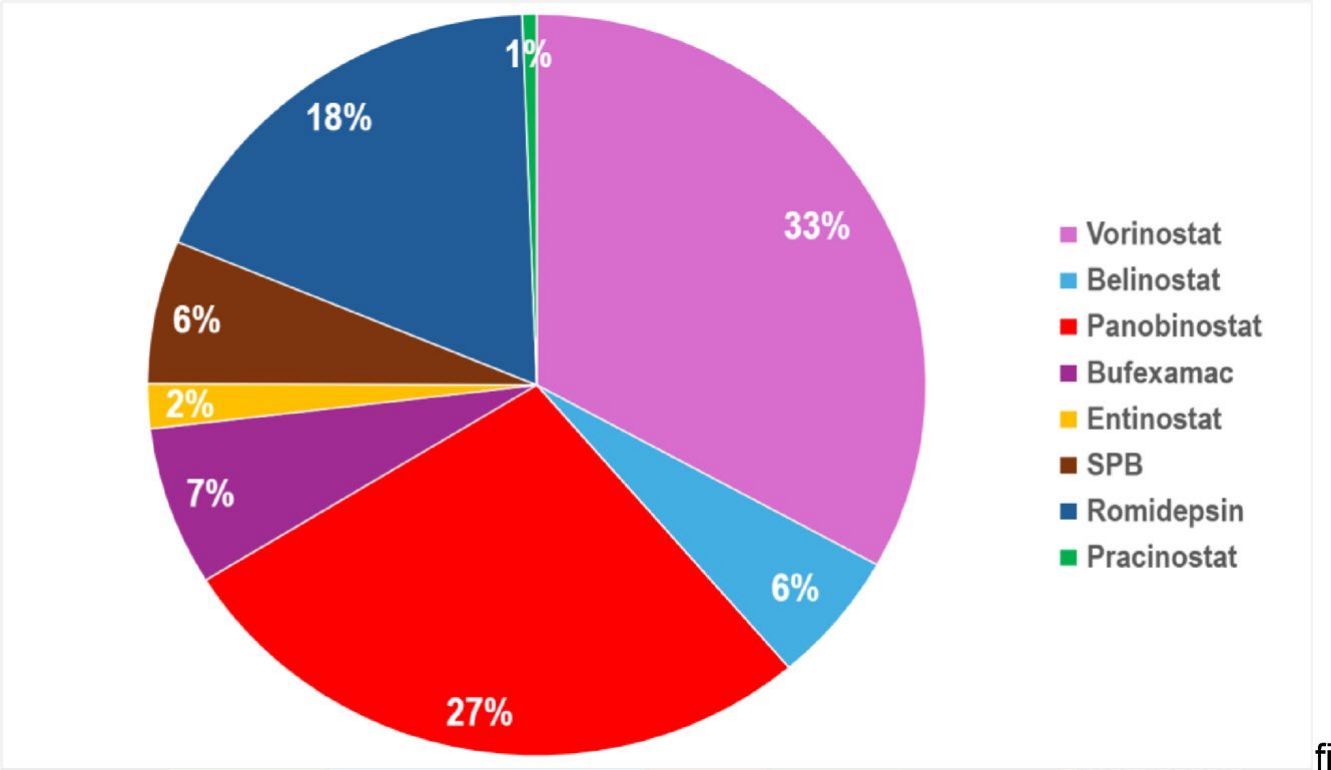
A pie chart displaying the percentage contribution of each HDAC inhibitor to the total number of ADRs, to the nearest whole number. The pie chart highlights the proportional representation of each HDACIs ADRs within the overall dataset. Full data, including absolute values for each SOC for each HDAC inhibitor are available in Table S5.

### ADRs

ADRs were found to be statistically significant across 15/27 SOCs (*p* <.05) for the HDACIs (Table S6). General disorders & administration site conditions had the highest number of total ADRs (*n* = 1,801), though not statistically significant. Vorinostat led with the most ADRs (~ 33% of all HDACIs ADRs) and provided high ADRs across the SOCs (*n* = 18/27) including GI disorders (*n* = 410), infections & infestations (*n* = 417) and blood & lymphatic disorders (*n* = 365), all reaching statistical significance (*P* <.05). Intriguingly, Fig. 2 highlights bufexamac had almost a six-fold increase in skin and subcutaneous tissue disorders compared to vorinostat (656 vs. 114 reports, respectively). Sodium phenylbutyrate was the only drug with any reports (*n* = 5) in the pregnancy, puerperium & perinatal conditions. Overall pracinostat had the least ADRs reported (*n* = 76), with reports of (*n* = 0) in 11 SOCs, accounting for only ~ 1% of total HDACIs ADRs.

A visual inspection of Figs. 2 and 3 highlights there were higher incidences of ADRs with the hydroxamic acids and cyclic peptides, compared to the benzamide and short-chain fatty acid derivatives (Figure S1). This was expected, as hydroxamic acid-based ZBGs typically have higher binding affinities for Zn^2+^ within the HDAC active site and can uniquely chelate Zn^2+^ in a bidentate manner, essentially the hydroxamic acid group being able to attach to the Zn^2+^ in two places resulting in a stronger bonding network^18,19^. The lack of selectivity can result in chelation with HDAC isoforms and other zinc-dependent metalloproteins (i.e. carbonic anhydrase or aminopeptidase) within the body, contributing to its ADR profile^62,63^. Similarly, cyclic peptides have more complex structures compared to benzamide and short-chain fatty acid derivatives (Figure S1), thus are more likely to interact extensively with surrounding amino-acids within the HDAC active site as well as other non-HDAC proteins in the body^15,64^.

## Discussion

### Blood and lymphatic system disorders

Vorinostat (*n* = 365), panobinostat (*n* = 323) and romidepsin (*n* = 286) had the highest related blood and lymphatic system ADRs (*P* <.05). Further analysis revealed (*n* = 125), (*n* = 142) and (*n* = 113) reports of thrombocytopenia with vorinostat, panobinostat and romidepsin, respectively. Given they all target HDAC1 and 2 at clinically achievable levels, their inhibition of HDAC1 and 2 is likely to contribute to the increase in thrombocytopenia seen with these HDACIs. Dual inhibition of HDAC1 and HDAC2 in mice models resulted in thrombocytopenia by causing megakaryocyte apoptosis; differentiation of megakaryocyte-erythrocyte lineages is crucial for producing red-blood cells and platelets^65^. These HDACIs, except for belinostat, demonstrated the highest V_d_ thus greater concentrations of the drug can get into the bone-marrow, enhancing their effect on megakaryocytes^66^.

Furthermore, only vorinostat, panobinostat and romidepsin showed potent, off-target interactions with the BRD4 protein. The inhibition of BRD4 is commonly associated with haematological ADRs, including thrombocytopenia^67^. Approximately, 15% of patients enrolled in the phase I study of the BRD4 inhibitor AZD5153 experienced grade 3 or higher thrombocytopenia, making it amongst the most reported haematological side effect^68^.

The HDACIs used for the treatment of blood cancers (Figs. 2 and 3) had higher incidences of ADRs. The lowest ADRs were recorded for entinostat (*n* = 21), sodium phenylbutyrate (*n* = 17) and bufexamac (*n* = 13), consistent with their primary indications being unrelated to the blood and lymphatic system. Therefore, the mechanism of HDAC1 and HDAC2 inhibition plus the off-target BRD4 inhibition shared by vorinostat, panobinostat and romidepsin specifically could explain the higher incidences of thrombocytopenia observed, despite also being used for haematological cancers^65,67,68^.

### Cardiac disorders

Vorinostat had the highest reports of cardiac ADRs (*n* = 146, *P* <.05), which was correlated to its potent inhibition of the human-ether-à-go-go-related gene (*h*ERG) ion-channel (IC_50_ = 320 nM vs. C_max_ = 1,200 nM). The *h*ERG ion-channels are vital for cardiomyocyte repolarisation, and thus inhibition of these channels has been associated with ventricular arrythmias, known as *torsades de pointes*^69–71^. Vorinostat was the only HDACI to report any cases of *torsades de pointes*, totalling (*n* = 7) further supporting the correlation between *h*ERG and the ADR profile.

### Musculoskeletal and connective tissue disorders

Higher incidences of musculoskeletal and connective tissue disorders were reported with vorinostat (*n* = 81, *P* <.05), potentially attributed to its potent inhibition of HDAC4 (IC_50_ = 540 nM vs. C_max_ = 1,200 nM). HDAC4 is crucial for skeletal muscle regeneration, mice with HDAC4 knock-out suffered perinatal deaths due to abnormal skeletal formation^72,73^.

### Pregnancy, puerperium & perinatal conditions

Sodium phenylbutyrate was the only HDACI with ADRs in this pregnancy-associated SOC (*n* = 5), though statistical significance could not be determined. Sodium phenylbutyrate is used for the treatment of urea cycle disorders, conditions that can affect anyone of any age, including women of child-bearing potential^74^. Pregnancy can be a trigger for urea cycle disorders, supporting the use of sodium phenylbutyrate in this patient group^75^. In contrast, most other HDACI are primarily used in oncology, where pregnancy is rare owing to patients ages, co-morbidities, and infertility from cancer treatments^76^. Sodium phenylbutyrate is also the only category C medication used during pregnancy, reflecting limited harm to the foetus whereas the other HDACIs studied are category D, suggesting confirmed foetal harm or lack sufficient research^21,50,51,77–79^. The fact that clinicians are likely to favour using category C drugs in practice and the demographic differences are likely to explain the observed pregnancy-associated ADRs being only associated with sodium phenylbutyrate.

### Psychiatric and nervous system disorders

Table 3 reveals entinostat as the only potent HDAC9 inhibitor, (IC_50_ = 0.5 nM vs C_max_ = 49 nM). Research shows that HDAC9 is expressed in the brain and HDAC9 inhibition can induce an anti-depressant like effect^80,81^. HDAC9 was found to be highly expressed in the hippocampal neurons of mice with chronic resistant stress and depression and concluded HDAC9 inhibition could be a promising treatment strategy for depressive disorders^80^. Hence, this may be part of the explanation for entinostat having a lack of ADR reports relating to anxiety, depression, or suicide, as a potential beneficial drug reaction (BDR). The other HDACIs studied, which did not inhibit HDAC9 at a clinically achievable level, were associated with at least (n ≥ 1) case of anxiety or depression, with vorinostat and panobinostat, respectively (n = 6 and n = 3 cases, respectively, *P* <.05). Vorinostat was the only HDACI to report a completed suicide. Whilst this could be associated with its ability to penetrate the BBB (meeting 5/7 requirements) coupled with its particularly low PPB (71%) where more ’free drug’ is available to cross the BBB increasing the risk of psychiatric side effects (Table 2), this finding likely involves various confounding factors including psychiatric co-morbidities and socio-environmental factors, warranting further study.

Vorinostat has also been detected at concentrations > 10 nM in the CSF of paediatric brain tumour patients^54^. Therefore, it has the potential to clinically inhibit fibroblast growth factor receptor 1 (FGFR1) (IC_50_ = 12 nM vs. C_max_ = 1,200 nM). FGFR1 is a protein essential for hippocampal neuron formation and required for long-term memory consolidation and learning^82,83^. Inhibition of FGFR1 could alter vital pathways regulated by FGF2, which helps to regulate anxiety and associated mood disorders, and interfering FGFR1 signalling predisposes patients to psychiatric disorders including anxiety and depression, which could potentially be another reason for the high incidences of psychiatric ADRs seen with vorinostat^84,85^.

### Skin and subcutaneous tissue disorders

Bufexamac led with the greatest ADRs relating to the skin and subcutaneous tissue (*n* = 656), with (*n* = 214) for contact dermatitis. Bufexamac was withdrawn from the EU market in 2010 due to concerns of allergic contact dermatitis, therefore the detection of this signal in the dataset further reinforces the validity of this approach to signal detection using the WHO VigiAccess database^21^. The aryl ether functional group unique to bufexamac (Fig. 2), has the potential to form quinone methide metabolites, which can cause skin irritation^86–88^. Coupled with being the only HDACI studied that is applied topically (Table 1), to treat inherently inflammatory skin conditions such as eczema likely explains this much higher incidence of skin ADRs observed^21^.

Vorinostat (*n* = 114), romidepsin (*n* = 67) and belinostat (*n* = 37) followed bufexamac with the highest ADRs in this SOC, and these 3 HDACIs are primarily used in cancers relating to the skin (Table 3). This patient population is already likely to have compromised skin integrity and other skin malignancies, which may predispose them to greater incidences of associated skin ADRs as a result. Thus, the confounder of indication prevented further mechanistic interpretation.

Specifically, vorinostat reported the highest cases of alopecia (*n* = 13), correlating to its potent inhibition of FGFR1, given alopecia is also a known ADR of FGFR1 inhibitors^89^. For example, 49% of patients treated with the FGFR inhibitor pembigatanib experienced grade 1 or 2 alopecia^90^. However, only (*n* = 2) cases were reported with romidepsin and sodium phenylbutyrate, respectively, despite having the next highest reports of alopecia, and no reports of alopecia were associated with the other HDACIs, none of which inhibited FGFR1. Thus, the almost 7-fold increase in alopecia cases observed with vorinostat, suggests FGFR1 inhibition may be contributing to this ADR, though this tentative finding requires further investigation to confirm this association.

### Infections and infestations

HDACIs are given to immunocompromised patients, who have likely undergone prior chemo or radiotherapy, as HDACIs are usually indicated in the later stages of cancers, when other treatment options have been exhausted^91^. This inherent vulnerability could explain the high incidences of infections seen across all the HDACIs, totalling (*n* = 1,048, *P* <.05), with the highest cases for vorinostat (*n* = 417), panobinostat (*n* = 289) and romidepsin (*n* = 180). Various HDAC isoforms participate in regulating immune responses; HDAC4 encourages type I IFN signalling by interacting with TBK1/IKKε to initiate antiviral immunity, HDAC5 regulates macrophage differentiation and recruitment and HDAC8 preserves the function of natural killer cells, though no clear patterns could be detected^92–94^.

### GI and site of administration disorders

Both GI and administration site disorders are common ADRs of HDACIs (*n* = 1,307 and *n* = 1,801, respectively). GI disorders were most pronounced for the acidic drugs; the hydroxamic acid derivatives and cyclic peptides, their acidic pKa could lead to irritation in the gastric mucosa^95^. Particularly, panobinostat had the greatest reports for GI disorders (*n* = 488, *P* <.05). Table 3 highlights that panobinostat is usually co-administered with the corticosteroid, dexamethasone, and proteasome inhibitor, bortezomib: both well-documented for causing GI bleeding and intestinal irritation^20,96,97^. Panobinostat was the most potent HDAC3 inhibitor (IC_50_ = 2.0 nM vs. C_max_ = 8.0 nM), an enzyme involved in preserving the intestinal epithelial barrier and mucosa integrity, which may explain the higher reports of GI ADRs with panobinostat^98^.

Both belinostat and romidepsin had the most administration site disorders compared to its reports within other SOCs (*n* = 151) and (*n* = 380), respectively. They are primarily administered *via* the intravenous route (Table 3), and the acidity of the drugs could cause irritation in the veins and surrounding tissue^99^.

### Neoplasms benign, malignant & unspecific

Romidepsin, the only pro-drug amongst the HDACIs studied to treat cancer, requires intracellular activation *via* glutathione or thioredoxin^100^. At tumour sites there can be reduced levels of glutathione or thioredoxin resulting in suboptimal activation of romidepsin and less effective treatment, reflected in the (*n* = 48) cases of malignant neoplasm progressions - the most significant amongst all the HDACIs studied (*P* <.05)^101,102^. This tentative observation emphasises the need for more research into the intracellular and biochemical activation of romidepsin in cancer cells, to ensure optimal treatment and reduction of cancer progression.

Healthcare professionals (HCPs) and staff are encouraged to report any ADRs to their respective pharmacovigilance jurisdiction which in turn feeds into the WHO VigiAccess database, though critically causality does not need to be proven^59^. Therefore, the HDACIs may not be associated with the ADR, but rather this may be due to other co-founders (i.e. polypharmacy, co-morbidities, genetics, and others). Typically, HDACIs are used in the advanced stages of cancer, thus patients are likely to have had prior treatment with chemo/radiotherapy or other platinum-based therapies which might have contributed to or caused the ADR^91^.

Fundamentally, the spontaneous reporting of ADRs has weaknesses, particularly the risk of under-reporting^103^. Many factors can impact the reporting of a drug; time on the market, ADR severity, and novelty and indication of the drug^104^. This is particularly important considering the small number of reports for HDACIs generally. Within this study, ADR reports varied through the years from 1996 to 2024, this coupled with the lack of prescribing data available makes it difficult to standardise the ADRs across the SOCs. Some of these drugs are still in clinical trials and bufexamac has been withdrawn from the market, therefore affecting the number of ADRs generated^21^.

Despite the pharmacology data being extracted from ChEMBL, it is unlikely it provided every protein interaction possible, therefore interactions with other targets remain unknown.

## Conclusion

The development of HDACIs has shown promise as an effective treatment strategy for many cancers, emphasising the need to understand their polypharmacology and associated ADRs. Vorinostat accounted for almost a third of the total ADRs (*n* = 4,225), with side effects ranging from cardiac, musculoskeletal, gastrointestinal, and psychiatric system organ classes. Vorinostat also had the most off-target interactions (*n* = 9), including the *h*ERG ion channel (IC_50_ = 322 nM vs. C_max_ = 1,200 nM) translating to the most cardiac ADRs, and the only HDACI with reports of *torsades de pointes*. Vorinostat also had the greatest cases of anxiety and depression, potentially ascribed to its lack of HDAC9 inhibition and ability to penetrate the BBB, in contrast entinostat, a potent HDAC9 inhibitor (IC_50_ = 0.5 nM vs. C_max_ = 49 nM), reported no cases of anxiety of depression. While vorinostat’s ability to uniquely inhibit HDAC4 (IC_50_ = 540 nM) this likely explains its contribution of approximately 40% of all HDACIs musculoskeletal ADRs, given its prominent role in skeletal myogenesis. Dual inhibition of HDAC1 and 2 may have contributed to vorinostat, panobinostat and romidepsin’s increased incidences of thrombocytopenia, alongside their higher V_d_’s, validating the existing link between increased V_d_ and thrombocytopenia.

Romidepsin activation as a pro-drug could explain its high incidences of neoplasms. Romidepsin and belinostat, which are administered i.v., showed the most administration site disorders within their own SOCs, but not across all the HDACIs studied, thus these tentative links require further investigation. No clear patterns emerged between the infection ADRs and polypharmacology, likely attributed to the overlapping roles of HDACs within the immune system.

The methodology used in this study was substantiated by reproducing ADR signals linked to HDACIs that were reported from clinical trials, specifically haematological (*n* = 1,090), infections and infestations (*n* = 1,048) and gastrointestinal abnormalities (*n* = 1,307). Integrating chemical and pharmacological data with real-life, global pharmacovigilance datasets provided valuable insights to investigate ADRs associated with HDACIs. The data generated by this powerful approach could be used to predict the ADR profiles of novel HDACIs coming onto the market in the years to come.

## Supplementary Information

Below is the link to the electronic supplementary material.

Supplementary Material 1

## Data Availability

Data is provided within the manuscript or supplementary information files.
