# Supplementary material for: Adverse drug reaction profiles of histone deacetylase inhibitors
Source: Sci Rep. 2025 Oct 14;15:35880. doi: 10.1038/s41598-025-19717-w (PMC12521396; doi:10.1038/s41598-025-19717-w)
Supplement: Supplementary file 1 — Supplementary Material 1 [file 41598_2025_19717_MOESM1_ESM.pdf]

SUPPORTING INFORMATION

Adverse Drug Reaction Profiles of Histone Deacetylase Inhibitors

Ruqayyah Begum,[1] Jason L. Parsons,[2] and Alan M. Jones[1*]

[1]School of Pharmacy, School of Health Sciences, College of Medicine and Health, University of Birmingham, Edgbaston, B15 2TT, United Kingdom.

[2] Department of Cancer and Genomic Sciences, University of Birmingham, Edgbaston,

Birmingham, B15 2TT, UK

Corresponding author: a.m.jones.2@bham.ac.uk

CONTENTS

Table S1	page 2
Table S2	page 3
Table S3	page 4
Figure S1	page 5
Table S4a	page 6
Table S4b	page 7
Table S5	page 8
Table S6	page 10

Classification Method	Description
**DoTS**	**Dose related:**<br>• Toxic effects -> From doses exceeding the usual therapeutic dose.<br>• Collateral effects -> From doses at the usual therapeutic dose.<br>• Hyper-susceptible -> From doses below the usual therapeutic dose, in susceptible patients.<br><br>**Time related:**<br>• *Independent* -> ADRs happen at any point during treatment.<br>• *Dependent*<br>→ Rapid reactions: take place if the drug is given too quickly.<br>→ Early reactions: take place soon after initiating treatment.<br>→ Intermediate reactions: take place after a delay.<br>→ Late reaction: take place after continued drug use or discontinuation.<br>→ Delayed reaction: take place long after drug exposure.<br><br>**Susceptibility:**<br>• Influenced by patient factors i.e., age, gender, genetic material, disease or changed physiology.
**Rawlins-Thompson**	• **Type A: (Augmented)** -> Predictable, common and dose-dependent.<br>• **Type B: (Bizarre)** -> Not predictable, uncommon, immune or idiosyncratic.<br><br>*Extended types:*<br>• **Type C: (Chronic)**<br>• **Type D: (Delayed)**<br>• **Type E: (End of use)**<br>• **Type F: (Failure)**

Table S1. The key features of the DoTS and the Rawlins-Thompson ADR classification systems. **Abbreviations;** DoTS; dose, time, susceptibility.

HDAC inhibitor	Regulatory Status	Approval Dates
**Vorinostat**	Licensed by the FDA and EMA	FDA (2006), EMA (2009)
**Belinostat**	Licensed by the FDA	FDA (2014)
**Panobinostat**	Licensed in Japan, FDA and EMA<br>Orphan status* in the United Kingdom<br><br>**An orphan drug is a medicine used to treat rare diseases, affecting <200,000 people.*	Japan (2015)<br>FDA (2015)<br>EMA (2015)<br>NICE guidelines (2015) for the orphan disease multiple myeloma
**Pracinostat**	In clinical trials	N.A.
**Entinostat**	In clinical trials	N.A.
**Romidepsin**	Licensed by the FDA	FDA (2009)
**Bufexamac**	Withdrawn	N.A.
**Sodium phenylbutyrate**	Licensed by the FDA, EMA	FDA (1996)<br>EMA (1999)<br>NICE guidelines (2013)

Table S2. Overview of the licensing status of the eight HDAC inhibitors studied. **Abbreviations**: FDA, U.S Food and Drug Agency; EMA, European Medicines Agency; NICE, National Institute for Health and Care Excellence; N.A., not applicable.

Drug	Chemical Structure	Indications	Route of Administration	Treatment Regimen
**Vorinostat**	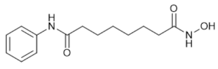	Cutaneous T-cell lymphoma	PO	400 mg OD
**Belinostat**	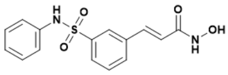	Refractory peripheral T-cell lymphoma	IV	1,000 mg/m^2 ,over 30 minutes QDS on Days 1–5 (treatment cycle is 21-days)
**Panobinostat**	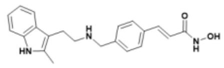	Multiple myeloma	PO	With bortezomib & dexamethasone
**Pracinostat**	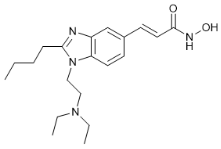	Acute myeloid leukaemia	PO	60 mg on 3 alternative days a week for 21 days. With 75 mg/m^2 of azacitidine
**Entinostat**	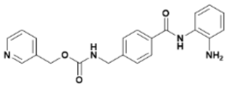	Hormone refractory breast cancer	PO	5 mg once a week. With 25 mg exemestane OD
**Romidepsin**	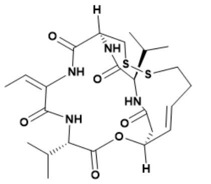	Cutaneous and peripheral T-cell lymphoma	IV	14 mg/m^2 on days 1, 8, and 15 (treatment cycle is 28 days)
**Bufexamac**	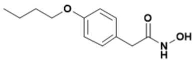	Skin conditions i.e. dermatitis or eczema	Topical	125-500 mg OD
**Sodium phenylbutyrate**	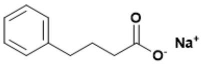	Urea cycle disorders	PO	9.9–13 g/m^2 OD

Table S3. Summary of the chemical structures, route of administrations, indication(s) and treatment regimens of the eight HDAC inhibitors studied. **Abbreviations**: PO, oral; IV, intravenous; OD, once a day, QDS, four times a day.

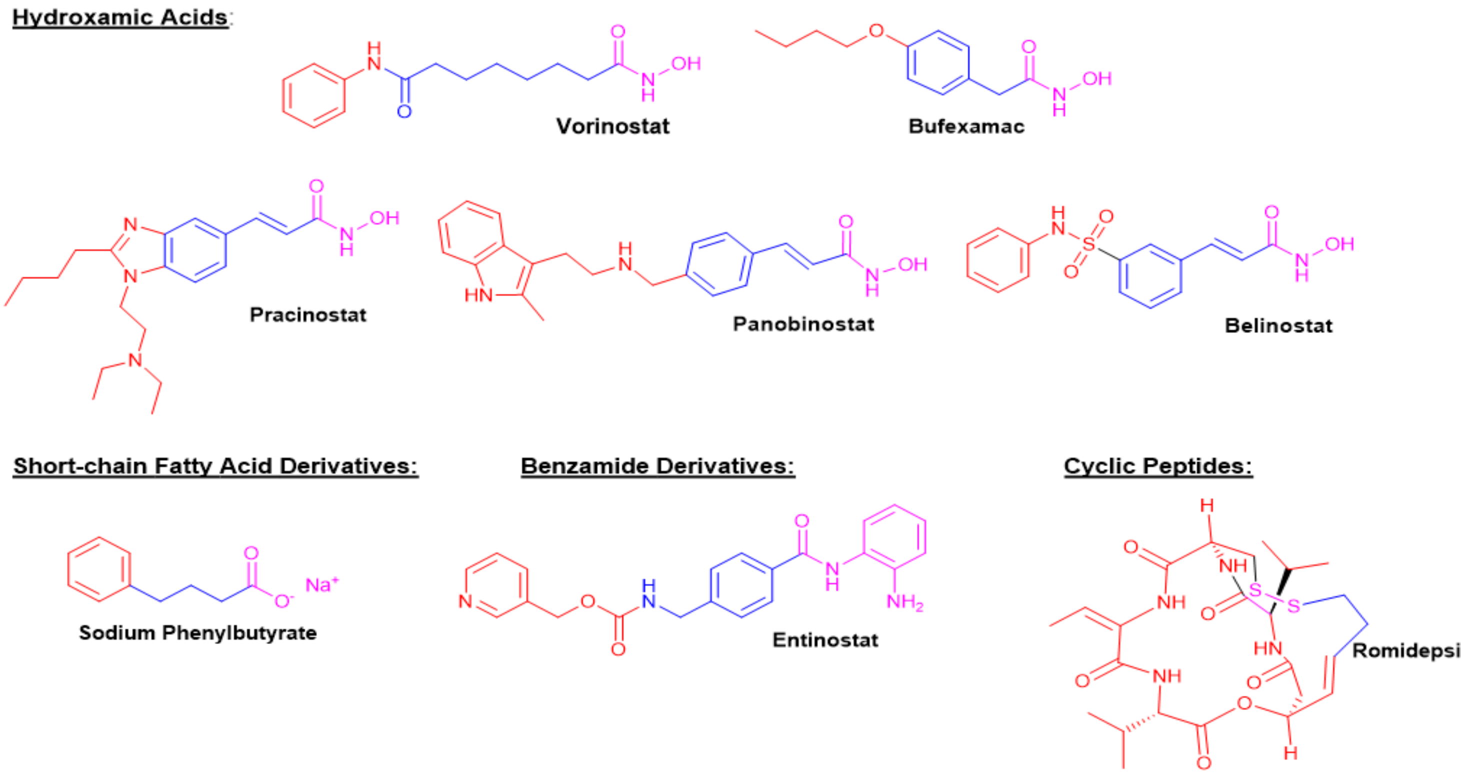

Figure S1. Chemical structures of the HDAC inhibitors studied. *Colour coding: red for cap group, blue for hydrophobic linker group and pink for zinc-binding group.*

Table S4a: HDAC inhibitors excluded from study, alongside the primary reason for exclusion.

HDAC inhibitor name	Primary reason for exclusion
Curcumin	Natural compound.
Levocarnitine	Amino acid.
Tucidinostat	<5 ADRs in every system organ class.
Mocetinostat	<5 ADRs in every system organ class.
Fimepimostat	<5 ADRs in every system organ class.
Ricolinostat	<5 ADRs in every system organ class.
Abexinostat	<5 ADRs in every system organ class.
Tefinostat	<5 ADRs in every system organ class.
Nanatinostat	<5 ADRs in every system organ class.
Tacedinaline	<5 ADRs in every system organ class.
Citarinostat	<5 ADRs in every system organ class.
Resminostat	<5 ADRs in every system organ class.
Dacinostat	<5 ADRs in every system organ class.
Tasquinimod	<5 ADRs in every system organ class.
Resminostat	<5 ADRs in every system organ class.
Sulforaphane	<5 ADRs in every system organ class.
Luteolin	<5 ADRs in every system organ class.

Table S4b: The known side effects of HDAC inhibitors, based on their Zinc-binding groups.

Hydroxamic acids	Benzamide	Carboxyl	Thiol
Thrombocytopenia	Thrombocytopenia	Thrombocytopenia	Thrombocytopenia
Anaemia	Anaemia	Teratogenic	Fatigue
Fatigue	Diarrhoea	Hepatoxicity	Infections
Vomiting	Headaches	Nausea	

Table S5: Summary of the suspected ADR profiles for the eight HDAC inhibitors studied using the WHO VigiAccess database, accessed 14.11.24 alongside the Chi-squared statistical results for each system organ class to 2 decimal places, where n<5 reports were present were excluded from the analysis. Highlighted yellow met statistical significance (p<0.05).

	**Vorinostat**	**Belinostat**	**Panobinostat**	**Pracinostat**	**Entinostat**	**SODIUM PHENYL BUTYRATE**	**Romidepsin**	**Bufexamac**	**Total ADRs**	**P-value**
**Blood & lymphatic system disorders**	365	45	323	20	21	17	286	13	1 090	<0.05
**Cardiac disorders**	146	22	106	3	6	11	94	3	391	<0.05
**Congenital, familial & genetic disorders**	2	0	0	0	0	2	1	0	5	n.d.
**Ear & labyrinth disorders**	6	3	3	0	0	1	8	0	21	0.99
**Endocrine disorders**	7	1	6	0	1	3	2	0	20	0.96
**Eye disorders**	14	4	10	0	3	4	15	8	58	0.90
**GI disorders**	410	70	488	10	22	116	184	7	1 307	<0.05
**General disorders & admin site conditions**	529	151	519	7	39	86	380	90	1 801	0.00
**Hepatobiliary disorders**	56	5	41	0	3	19	38	1	163	<0.05
**Immune system disorders**	24	4	10	1	2	2	33	28	104	0.09
**Infections & infestations**	417	52	289	7	33	50	180	20	1 048	<0.05
**Injury, poisoning & procedural complications**	304	33	164	4	3	64	112	3	687	<0.05
**Investigations**	383	60	573	3	14	66	300	5	1 404	n.d.
**Metabolism & nutrition disorders**	270	41	174	4	20	128	93	3	733	<0.05
**Musco skeletal & connective tissue disorders**	81	25	58	1	12	9	24	5	215	<0.05
**Neoplasms benign, malignant & unspecified**	183	25	177	5	12	2	200	0	604	<0.05

**Nervous system disorders**	231	36	177	3	14	72	88	23	644	<0.05
**Pregnancy, puerperium & perinatal conditions**	0	0	0	0	0	5	0	0	5	n.d.
**Product issues**	6	1	1	0	0	18	4	0	30	0.31
**Psychiatric disorders**	76	17	44	1	6	25	23	3	195	<0.05
**Renal & urinary disorders**	129	12	102	1	5	15	35	1	300	<0.05
**Reproductive system & breast disorders**	8	2	0	0	0	4	8	2	24	1.00
**Respiratory, thoracic & mediastinal disorders**	271	45	108	5	19	30	92	6	576	<0.05
**Skin & SC tissue disorders**	114	37	25	1	4	23	67	656	927	0.00
**Social circumstance**	4	1	2	0	0	8	1	0	16	n.d.
**Surgical & medical procedures**	30	5	26	0	0	13	5	1	80	<0.05
**Vascular disorders**	159	35	74	0	9	6	40	8	331	<0.05
**Total ADRs**	4225	732	3500	76	248	799	2313	886	12 779	0.00

Table S6: Calculations for the chi squared analysis for the eight HDAC inhibitors studied to 2 d.p.

System Organ Class	Vorinostat	Belinostat	Panobinostat	Pracinostat	Entinostat	SODIUM PHENYLBUTYRATE	Romidepsin	Bufexamac	Average	X^2
**Blood & lymphatic system ADRs**	385	45	323	20	21	17	286	13	138.75	<0.05
**Cardiac ADRs**	146	22	106	3	6	11	94	3	64.16666667	<0.05
**Congenital, familial & genetic ADRs**	2	0	0	0	0	2	1	0	All data excluded as ≤ 5	
**Ear & labyrinth ADRs**	6	3	3	0	0	1	8	0	7	0.99
**Endocrine ADRs**	7	1	6	0	1	3	2	0	6.5	0.96
**Eye ADRs**	14	4	10	0	3	4	15	8	11.75	0.90
**GI ADRs**	410	70	488	10	22	116	184	7	185.71	<0.05
**General disorders & admin site ADRs**	529	151	519	7	39	86	380	90	225.13	0
**Hepatobiliary ADRs**	56	5	41	0	3	19	38	1	31.8	<0.05
**Immune system ADRs**	24	4	10	1	2	2	33	28	23.75	0.09
**Infections & infestations**	417	52	289	7	33	50	180	20	131	<0.05
**Injury, poisoning & procedural complications**	304	33	164	4	3	64	112	3	135.4	<0.05
**Investigations**	383	60	573	3	14	66	300	5	200.14	0
**Metabolism & nutrition ADRs**	270	41	174	4	20	128	93	3	121	<0.05
**Musculoskeletal & connective tissue ADRs**	81	25	58	1	12	9	24	5	30.57	<0.05
**Neoplasms benign, malignant & unspecified**	183	25	177	5	12	2	200	0	100.33	<0.05
**Nervous system ADRs**	231	36	177	3	14	72	88	23	91.57	<0.05
**Pregnancy, puerperium & perinatal conditions**	0	0	0	0	0	5	0	0	Only 1 data point ≥5	n.d.
**Product issues**	6	1	1	0	0	18	4	0	12	0.31
**Psychiatric disorders**	76	17	44	1	6	25	23	3	31.83	<0.05
**Renal & urinary ADRs**	129	12	102	1	5	15	35	1	49.67	<0.05
**Reproductive system & breast ADRs**	8	2	0	0	0	4	8	2	8	1
**Respiratory, thoracic & mediastinal ADRs**	271	45	108	5	19	30	92	6	72	<0.05
**Skin & SC tissue ADRs**	114	37	25	1	4	23	67	656	153.67	0
**Social circumstances**	4	1	2	0	0	8	1	0	Only 1 data point ≥5	n.d.
**Surgical & medical procedures**	30	5	26	0	0	13	5	1	15.80	<0.05
**Vascular ADRs**	159	35	74	0	9	6	40	8	47.29	<0.05
